# High-Intensity Interval and Aerobic Training Alleviate Cardiac Pathology, Apoptosis, and Atrial Fibrillation in Rats with Chronic Kidney Disease: The Roles of FGF23 and Klotho

**DOI:** 10.3390/biom16040513

**Published:** 2026-03-30

**Authors:** Sina Rokhsati, Nazanin Shahsavari, Shahram Rabbani, Katsuhiko Suzuki, Kayvan Khoramipour

**Affiliations:** 1 Department of Exercise Physiology, Faculty of Physical Education and Sport Sciences, University of Tehran, Tehran 1417935840, Iran; sina.rokhsati@gmail.com (S.R.); nz.shahsavari@gmail.com (N.S.); 2 Research Center for Advanced Technologies in Cardiovascular Medicine, Tehran University of Medical Sciences, Tehran 1416634793, Iran; sh-rabbani@tums.ac.ir; 3 Faculty of Sport Sciences, Waseda University, Tokorozawa 359-1192, Japan; 4 i+HeALTH Strategic Research Group, Department of Health Sciences, Miguel de Cervantes European University (UEMC), 47012 Valladolid, Spain

**Keywords:** mineral metabolism, exercise, parathyroid hormone, CKD, FGF23, Klotho

## Abstract

Chronic kidney disease (CKD) leads to metabolic and cardiovascular complications, and the dysregulation of key biomolecules, namely fibroblast growth factor 23 (FGF23) and Klotho, plays a central role. This study investigated the effects of high-intensity interval training (HIIT) and moderate aerobic training (AT) on FGF23, Klotho, mineral metabolism, apoptosis markers (BAX, Bcl2), and atrial fibrillation (AF) in a rat CKD model. The study used 35 Wistar rats randomly assigned to control (CTL), sham (SH), CKD, CKD + HIIT, and CKD + AT groups. CKD was induced by 5/6 nephrectomy surgery. Exercise interventions consisted of eight weeks of HIIT (80–100% of maximum speed, 24–54 min/week) or AT (45–55% of maximum speed, 40–60 min/week), conducted three times weekly on a treadmill. We measured heart weight, blood levels of FGF23, Klotho, and mineral metabolism markers, as well as the heart expression of apoptosis proteins (i.e., BAX, Bcl2) and atrial fibrillation (AF). Both exercise types reduced the heart weight and heart/body weight ratio; attenuated CKD-induced elevations in FGF23 and reductions in Klotho; improved blood levels of phosphate, PTH, and vitamin D; and modulated apoptotic markers by decreasing BAX and increasing Bcl2 levels. Exercise improved cardiac function and reduced the AF duration. These findings emphasize that exercise could be a helpful non-pharmacological intervention to ameliorate CKD-induced cardiovascular and metabolic disturbances through the modulation of the FGF23 and Klotho pathways.

## 1. Introduction

Chronic kidney disease (CKD) is a major public health problem in which patients experience not only decreased kidney function but also a range of related pathological processes [1]. These interconnected conditions could also worsen each other [2]. One of the leading causes of mortality in CKD patients is cardiovascular disease [2], with atrial fibrillation (AF) as the most common [1]. Studies have shown that the prevalence of atrial fibrillation in CKD patients is 10–20% in non-dialysis patients and over 20–30% in end-stage renal disease (2). AF is a supraventricular cardiac arrhythmia induced by irregular excitation of the atrial muscle cells. Consequently, the atria cannot fully pump blood to the ventricles, and the ventricular beats are not in line with the atrial rhythm [3].

Fibroblast growth factor 23 (FGF23), a sensitive biomarker for impaired phosphate regulation in kidney patients, could be a driver of CKD-induced AF [4]. The circulating levels of this hormone increase in response to declining kidney function, promoting phosphate excretion into the urine and compensating for persistent phosphorus retention [5]. Increased FGF23 could reduce the production of the active form of vitamin D in the kidneys and increase the secretion of parathyroid hormone (PTH) [6], resulting in greater vascular calcification, cardiac hypertrophy, and the development of AF [5]. Research has shown that increasing levels of FGF23 and reduced levels of the active form of vitamin D could play important roles in increasing mortality in CKD patients [7,8,9].

In this context, the protein Klotho could also play a regulatory role by modulating FGF23 activity [10]. Klotho is a transmembrane protein that acts as a co-receptor for FGF23 and facilitates its binding to FGF receptors [10]. Klotho deficiency disrupts this regulatory axis and exacerbates the adverse cardiovascular effects of elevated FGF23 [11]. Specifically, reduced Klotho levels enhance vascular calcification, promote cardiac remodeling, and increase the risk of AF [12]. Restoring Klotho function can attenuate FGF23-mediated damage, protect the heart from post-injury remodeling, and improve overall cardiovascular function in CKD [13].

Evidence has shown that aerobic exercise can reduce FGF23 levels and, by decreasing the left ventricular thickness, lower the risks of AF and cardiac hypertrophy. However, some studies have found these effects to be non-significant [14]. A 2024 meta-analysis of four randomized control trials in CKD patients (272 participants) found that physical exercise significantly decreased FGF23 levels (MD: −102.07 pg/mL, 95% CI: −176.23 to −27.91, *p* = 0.008) [14]. In contrast, a 2019 rat study showed that acute moderate-intensity exercise (30 min at 20 m/min for 3–15 days) had no significant effect on FGF23 gene expression in heart tissue [15]. These inconsistencies may be attributed to differences in the exercise modality, intensity, and intervention duration, which highlights the need for the present study [14]. A systematic review and meta-analysis including 12 studies involving 621 participants concluded that chronic exercise training (at least 12 weeks) can increase Klotho levels. Increased Klotho levels are associated with improved endothelial function, reduced oxidative stress, and enhanced vascular health [16]. The type of exercise is the crucial factor in determining the effect of exercise on Klotho expression [17].

Despite these findings, comprehensive studies on the effects of high-intensity interval training (HIIT) and moderate-intensity exercise on FGF23, Klotho, and AF in CKD are still lacking. Investigating this relationship may help to identify effective non-pharmacological strategies for preventing and treating cardiovascular complications in patients with CKD. The present study aimed to examine the effects of HIIT and moderate-intensity continuous training on FGF23, Klotho, and AF in experimentally induced CKD rats. HIIT and moderate-intensity continuous training were selected to compare distinct cardiovascular loading patterns in CKD rats. HIIT delivers brief near-maximal hemodynamic stress for potent endothelial gains, while moderate-intensity continuous training applies steady, moderate loads for sustained endurance adaptations.

CKD can be viewed as a systemic disorder that can disrupt mineral metabolism, leading to cardiovascular remodeling. The FGF23–Klotho axis can be considered as a central mechanistic pathway linking renal dysfunction to cardiovascular complications, particularly AF, which can be affected by various kinds of exercise.

## 2. Materials and Methods

### 2.1. Animals and Ethical Issues

In this study, 35 male Wistar rats, weighing 250–300 g, were purchased from the Pasteur Institute of Tehran, Iran. The animals were kept under a standard 12:12 light–dark cycle with a temperature of 22 ± 2 °C and relative humidity of 55 ± 5%. All animals had free access to water and food. All laboratory procedures and protocols were approved by the Ethics Committee of the University of Tehran (ethics code: IR.UT.SPORT.REC. 1398.018, 25 May 2019). All study procedures were conducted in compliance with the ARRIVE 2.0 guidelines and clarified humane endpoints and analgesia use. No animals died during the surgical procedure, recovery period, or exercise intervention. Likewise, no animals met the predefined humane endpoint or exclusion criteria (e.g., surgical complications, inability to recover from anesthesia, or failure to complete the training protocol). Animals were randomly (i.e., using a computer-based random number generator) divided into five groups as follows (*n* = 7 in each group): control (CTL), sham (SH), CKD, CKD + HIIT, and CKD + aerobic training (CKD + AT). An a priori power analysis was performed using the G*Power software (version 3.1.9.7), assuming an effect size of 0.8, a significance level (α) of 0.05, and statistical power of 80% for detecting differences among groups using one-way ANOVA. The analysis indicated that a minimum of eight animals per group was required. There was no drop in the number of animals during the study. Potential confounders were minimized by standardizing the housing conditions, handling procedures, and measurement protocols across groups. The order of outcome measurements was randomized, and animals from different treatment groups were interspersed spatially within the housing facility to reduce location-related bias. The researchers who performed the animal work and statistical analysis were blind to the grouping.

### 2.2. Surgical Procedures

CKD was induced by two-stage 5/6 nephrectomy performed two weeks apart: left kidney surgery on day 0 followed by right kidney surgery on day 14, with daily monitoring during the 2-week recovery period between stages; no perioperative mortality occurred. In the first stage (day 0), a partial nephrectomy was performed on the left kidney, in which approximately two-thirds of the renal mass (upper and lower poles) was removed. In the second stage (day 14), the same procedure was performed on the right kidney.

The animals in the CKD, CKD + HIIT, and CKD + AT groups were anesthetized by the intraperitoneal injection of 50 mg ketamine and 5 mg xylazine per kg of body weight. The surgical procedure followed the instructions from a previous study [18] in which the upper- and lower-third parts of the kidney were resected. For nephrectomy, the abdominal area was incised along the linea alba to a length of 3 cm, and the left kidney was exposed. The kidney capsule, surrounding adipose tissue, and renal fascia were then removed. The upper- and lower-third parts of the kidney were closed with a 3-0 silk surgical suture. One-third of the kidney was then removed close to the ligated area, and both sides of the kidney were covered with a layer of oxidized cellulose to prevent bleeding. To prevent infection, cefazolin was administered intramuscularly at a dose of 20 mg/kg. The abdominal muscles and skin were then sutured with Vicryl 3/0. Post-surgery analgesia was administered using buprenorphine (0.05 mg/kg, subcutaneously) immediately after surgery and every 12 h for 48 h to minimize postoperative pain, in accordance with institutional animal care guidelines. After two weeks of recovery and relative adaptation, the same procedure (i.e., removal of two-thirds of the renal mass) was performed on the right kidney. In the SH group, the same treatment was conducted without sectioning the kidney, and these animals served as the surgical group. Animals were monitored daily during the recovery period for surgical wound healing, general behavior, and signs of postoperative complications. No perioperative mortality or surgical complications occurred during the study.

### 2.3. Training Program

Two weeks after surgery, animals in the CKD + HIIT and CKD + AT groups began the exercise protocols. The exercise programs were conducted in three sessions per week for eight weeks. Rats walked at a slow pace (5 m/min for 5 min) for two weeks to familiarize them with the treadmill (Iranian Danesh Salar, Tehran, Iran) and exercise training. The training groups performed warm-up and cool-down periods at the beginning and end of the 10 min exercise at a speed of 8 m/min. The details of the exercise programs are presented in Table 1 and Table 2. Vmax was determined by performing incremental tests. The test protocol started at 6 m/min for 2 min, with the speed increasing by 2 m/min every 2 min until exhaustion. Exhaustion was defined as the inability to maintain a running pace despite gentle prodding, with animals falling to the rear of the treadmill three consecutive times. We did not use electrical stimulation during animal training [19].

### 2.4. Electrocardiogram and Echocardiography

Surface and bipolar ECGs (leads II, V1) were recorded under thiopental sodium anesthesia (50 mg/kg IP) using PowerLab^®^ (ADInstruments, Bella Vista, NSW, Australia) at a 2 kHz sampling rate for 5 continuous minutes, 48 h after the final training session. Baseline heart rates averaged 180–200 bpm under anesthesia (vs. 300–400 bpm in conscious rats). Data analysis in LabChart Pro v8 used automated peak detection (0.1 mV threshold, manually verified), with the P-wave duration measured from onset to end at a 50% amplitude across 10 consecutive cycles pre-/post-pacing.

AF was assessed two weeks after surgery and 48 h after the last training session by a blinded cardiologist. AF inducibility testing followed baseline recording. In this model, AF was not spontaneous; it was induced by programmed atrial burst pacing (3 s trains, 200–350 bpm in 10 Hz increments via esophageal electrodes), and AF susceptibility was quantified as the duration of pacing-induced AF episodes. AF was defined as sustained irregular atrial tachyarrhythmia (>30 s duration) with (1) atrial rates > 600 bpm, (2) absent P-waves, (3) R-R variation > 50 ms. Post-pacing P-wave prolongation (>120 ms) served as the primary AF burden metric, validated in rat CKD models where a high baseline HR hinders R-R analysis. Diagnosis was confirmed by dual-blinded investigators (inter-rater κ = 0.87), consistent with validated rat CKD-AF models.

### 2.5. Measurement of Biochemical Factors

Blood samples were obtained from the hearts of rats before they were sacrificed, and they were collected in normal tubes. Afterward, samples were centrifuged at 10,000 rpm at 4 °C for 10 min. The resulting serum samples were stored at −80 °C until analysis. The serum levels of calcium and phosphate were measured by spectrophotometric assays, using reagents and standards purchased from Thermo Fisher Scientific Inc. (Waltham, MA, USA). Calibration curves for spectrophotometric assays were constructed using standard solutions provided by the manufacturer, and linearity and recovery were confirmed according to standard protocols. Parathyroid hormone (PTH) and vitamin D were measured with radioimmunoassay kits (Cobas, Roche, Mannheim, Germany), according to the manufacturer’s instructions. Serum levels of FGF23 were determined by an ELISA kit (MyBioSource, MBS2515950, San Diego, CA, USA) with a coefficient of variation of less than 10%, according to the manufacturer’s recommendations. The serum concentration of Klotho was assessed by an ELISA kit (MyBioSource, MBS2885116, San Diego, CA, USA) with an intra-assay coefficient of variation of <1.5% and an inter-assay coefficient of <4.9%. Heart tissue was extracted and homogenized in ice-cold lysis buffer and centrifuged (12,000× *g*, 4 °C) to remove debris, and the supernatants were used for ELISA analysis of BAX and Bcl2, according to the manufacturer’s instructions. The heart expression of BAX was measured using an ELISA kit (MyBioSource, catalog number MBS701787) with an intra-assay coefficient of variation of less than 8% and inter-assay coefficient of variation below 10%. Bcl2 heart levels were quantified by an ELISA kit (MyBioSource, catalog number MBS287654) exhibiting an intra-assay CV under 6% and inter-assay CV below 9%.

All assays were performed in duplicate, and the mean values were used for analysis.

### 2.6. Statistical Analysis

The obtained values were analyzed by GraphPad Prism version 8 (GraphPad Software, San Diego, CA, USA). The data were expressed as the mean and standard error of the mean (mean ± SEM). The Shapiro–Wilk and Levene tests were applied to examine normality and homogeneity of variance, respectively. One-way analysis of variance (ANOVA) was employed to determine significant differences between the values of the experimental groups, followed by Tukey’s post hoc test. The level of statistical significance was set at *p* < 0.05.

## 3. Results

The Vmax values of the two training groups across various weeks are provided in Supplementary Table S1.

The results of the Shapiro–Wilk and Levene tests confirmed the normality and homogeneity of variance for all variables (Supplementary Table S2).

### 3.1. Body Weight, Heart Weight, and Their Ratio

**Figure 1:**
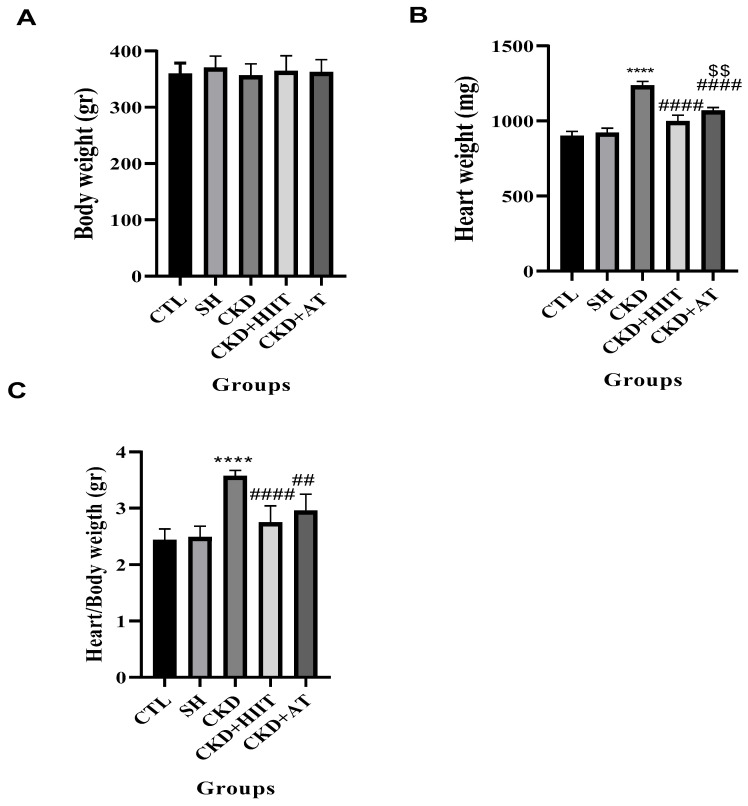
Effects of chronic kidney disease and high-intensity interval training on body weight, heart weight, and heart/body weight ratio. (**A**) Body weight, (**B**) heart weight, and (**C**) heart/body weight ratio in experimental groups. Data were analyzed using one-way ANOVA followed by Tukey’s post hoc test. Values are expressed as the mean ± standard error of the mean (*n* = 7), with *p* < 0.05 considered statistically significant. **** Significantly different vs. CTL and SH (*p* < 0.0001); #### significantly different vs. CKD (*p* < 0.0001); ## significantly different vs. CKD (*p* < 0.01); $$ significantly different vs. CKD + AT (*p* < 0.01). Abbreviations: CTL, control; SH, sham; CKD, chronic kidney disease; HIIT, high-intensity interval training; AT, aerobic training.

There was no significant difference in body weight between groups (F (4, 30) = 0.3780, *p* = 0.8225). However, a significant difference was observed for the heart weight (F (4, 30) = 27.63, *p* < 0.0001) and heart/body weight between groups (F (4, 30) = 15.36, *p* < 0.0001). While CKD showed a higher heart weight and heart/body weight compared to the SH and CTL groups (*p* < 0.0001), the CKD + HIIT and CKD + AT groups showed lower heart weights and heart/body wights compared to CKD (*p* < 0.0001). We observed a significant difference between the CKD + HIIT and CKD + AT groups in terms of heart weight (*p* < 0.01), but this difference was not significant for heart/body weight (*p* = 0.4546) (Figure 1).

### 3.2. FGF23 and Klotho

**Figure 2:**
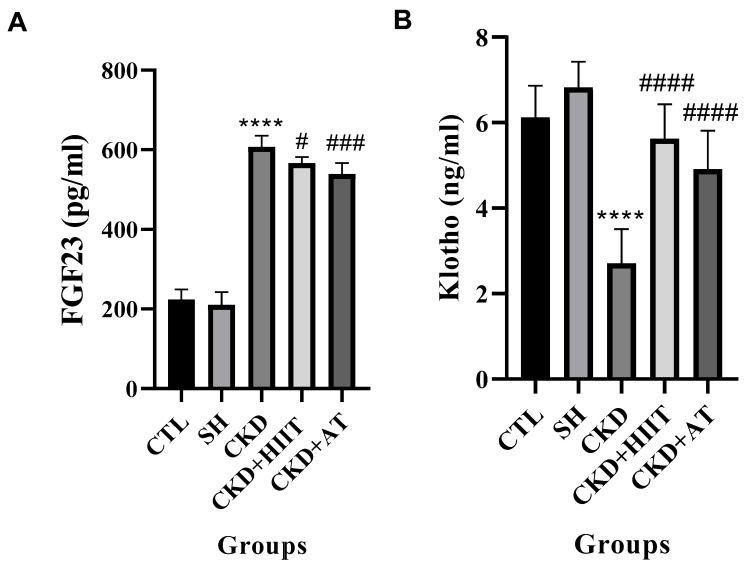
Effects of chronic kidney disease and high-intensity interval training on FGF23 and Klotho levels in serum. (**A**) FGF23, (**B**) Klotho. Data were analyzed using one-way ANOVA followed by Tukey’s post hoc test. Values are expressed as the mean ± standard error of the mean (*n* = 7), with *p* < 0.05 considered statistically significant. **** Significantly different vs. CTL and SH (*p* < 0.0001); #### significantly different vs. CKD (*p* < 0.0001); # significantly different vs. CKD (*p* < 0.05); ### significantly different vs. CKD (*p* < 0.001). Abbreviations: CTL, control; SH, sham; CKD, chronic kidney disease; HIIT, high-intensity interval training; AT, aerobic training.

The one-way ANOVA showed significant differences between groups in the FGF23 (F (4, 30) = 385.6, *p* < 0.0001) and Klotho (F (4, 30) = 28.82, *p* < 0.0001) levels in the serum. CKD showed higher levels of FGF23 (*p* < 0.0001) and lower levels of Klotho (*p* < 0.0001) compared to the CTL and SH groups. The CKD + HIIT group had lower levels of FGF23 (*p* < 0.05) and higher levels of Klotho (*p* < 0.0001) compared to the CKD group. Moreover, the CKD + AT group had higher levels of FGF23 (*p* < 0.001) and lower levels of Klotho (*p* < 0.0001) compared to the CKD group. There was no significant difference between the CKD + HIIT and CKD + AT groups in terms of FGF23 and Klotho levels (*p* ≥ 0.05) (Figure 2).

### 3.3. Vitamin D, Phosphate, Calcium, and PTH

**Figure 3:**
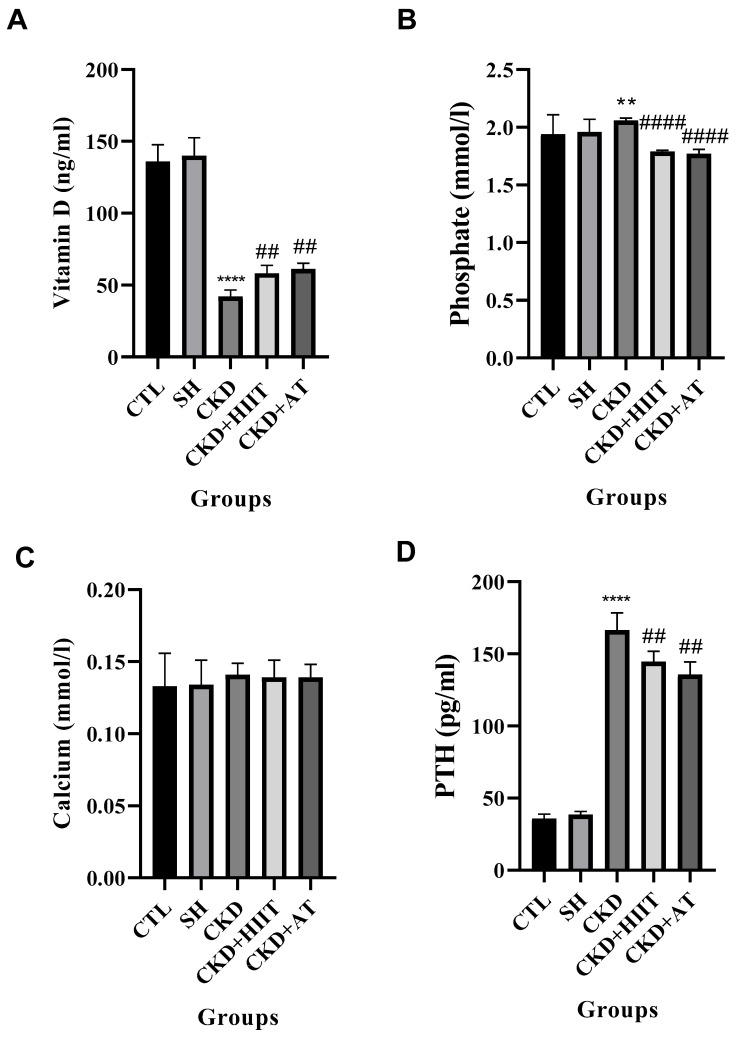
Effects of chronic kidney disease and high-intensity interval training on (**A**) vitamin D, (**B**) phosphate, (**C**) calcium, and (**D**) PTH in serum. Data were analyzed using one-way ANOVA followed by Tukey’s post hoc test. Values are expressed as the mean ± standard error of the mean (*n* = 7), with *p* < 0.05 considered statistically significant. **** Significantly different vs. CTL and SH (*p* < 0.0001); ** significantly different vs. CTL and SH (*p* < 0.01); #### significantly different vs. CKD (*p* < 0.0001); ## significantly different vs. CKD (*p* < 0.01). Abbreviations: CTL, control; SH, sham; CKD, chronic kidney disease; HIIT, high-intensity interval training; AT, aerobic training.

Significant differences were observed in the serum levels of vitamin D (F_4, 30_ = 54.38, *p* < 0.001), phosphate (F_4, 30_ = 12.15, *p* < 0.001), and PTH (F_4, 30_ = 23.51, *p* < 0.0001) but not the serum levels of calcium (F_4, 30_ = 8.36, *p* ≥ 0.05) between groups. The CKD group showed significantly lower levels of vitamin D compared with the SH (*p* < 0.0001), CTL (*p* < 0.0001), CKD + HIIT (*p* < 0.01), and CKD + AT (*p* < 0.01) groups. Furthermore, the blood levels of phosphate and PTH were higher in the CKD group compared with the CTL (*p* < 0.01 and *p* < 0.0001), CKD + HIIT (*p* < 0.0001 and *p* < 0.01), and CKD + AT (*p* < 0.0001 and *p* < 0.01) groups (Figure 3).

### 3.4. BAX, Bcl2, and BAX/Bcl2

**Figure 4:**
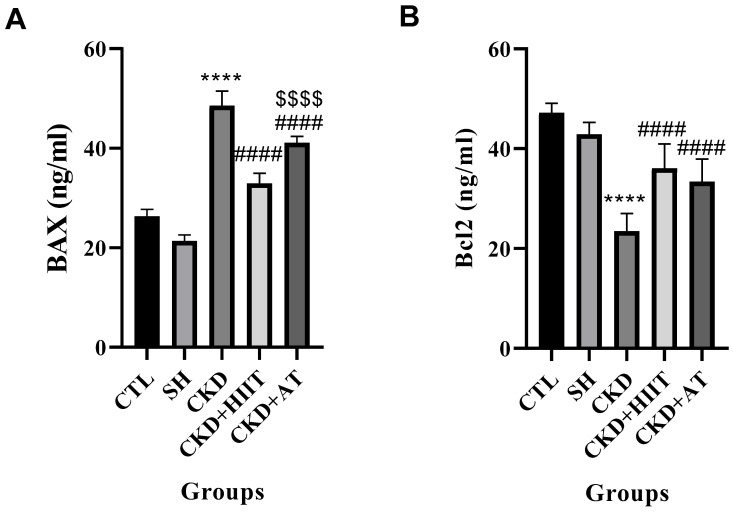
Effects of chronic kidney disease and high-intensity interval training on (**A**) BAX and (**B**) Bcl2 expression in the heart. Data were analyzed using one-way ANOVA followed by Tukey’s post hoc test. Values are expressed as the mean ± standard error of the mean (*n* = 7), with *p* < 0.05 considered statistically significant. **** Significantly different vs. CTL and SH (*p* < 0.0001); #### significantly different vs. CKD (*p* < 0.0001); $$$$ significantly different vs. CKD + AT (*p* < 0.001). Abbreviations: CTL, control; SH, sham; CKD, chronic kidney disease; HIIT, high-intensity interval training; AT, aerobic training.

Significant between-group differences were revealed by the ANOVA for BAX (F_4, 30_ = 33.69, *p* < 0.001), Bcl2 (F_4, 30_ = 27.61, *p* < 0.001), and Bcl2/BAX (F_4, 30_ = 45.36, *p* < 0.001). The results of Tukey’s post hoc test showed significantly higher levels of BAX for the CKD group compared with the CTL, SH, CKD + HIIT, and CKD + AT groups (*p* < 0.0001). In addition, the CKD + AT group showed higher levels of BAX expression compared with the CKD + HIIT group (*p* < 0.0001). The Bcl2 level was also lower in CKD compared with the CTL, CKD + HIIT, and CKD + AT groups (*p* < 0.0001) (Figure 4).

### 3.5. P-Wave Duration

**Figure 5:**
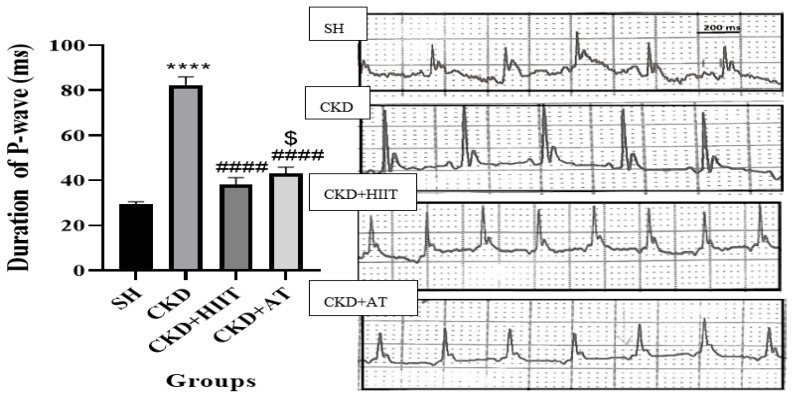
Baseline P-wave duration (pre-pacing) under thiopental anesthesia (HR 180–200 bpm). Representative ECG traces from each group; pacing-induced AF burden quantified separately via post-pacing P-wave prolongation. Data were analyzed using one-way ANOVA followed by Tukey’s post hoc test. Values are expressed as the mean ± standard error of the mean (*n* = 7), with *p* < 0.05 considered statistically significant. **** Significantly different vs. CTL and SH (*p* < 0.0001); #### significantly different vs. CKD (*p* < 0.0001); $ significantly different vs. CKD + AT (*p* < 0.05). Abbreviations: SH, sham; CKD, chronic kidney disease; HIIT, high-intensity interval training; AT, aerobic training.

The ANOVA showed significant differences between groups (F_4, 30_ = 27.61, *p* < 0.001). The P-wave duration was higher in the CKD group compared with the CTL, SH, CKD + HIIT, and CKD + AT groups (*p* < 0.001). In addition, the P-wave duration was lower in the CKD + HIIT group compared with the CKD + AT group (*p* < 0.05) (Figure 5).

## 4. Discussion

Our results showed that, while CKD increased the heart weight and heart/body weight ratio, both types of exercise reduced these measures, with a significantly lower heart weight observed in the CKD + HIIT group. In addition, CKD led to an increase in the serum levels of FGF23 and a decrease in the serum levels of Klotho; however, both AT and HIIT reversed these changes, with no significant difference between the interventions. The levels of vitamin D and PTH were significantly decreased and increased, respectively, in CKD compared with the CTL and SH groups, but both types of exercise reversed these changes, again with no significant difference between them. Furthermore, the heart expression of BAX was increased and that of Bcl2 decreased in CKD compared with the CTL and SH groups. BAX levels were lower in the CKD + HIIT and CKD + AT groups compared with the CKD group, with the lowest levels in the CKD + HIIT group. Both CKD + HIIT and CKD + AT showed higher levels of Bcl2, with no significant difference between them. The P-wave duration was also higher in the CKD group compared with the SH group but lower in both exercise groups compared with CKD, with the lowest level in the CKD + HIIT group.

The main link between CKD and AF is FGF23 and Klotho, with phosphate and calcium imbalance as central mediators of FGF23 and Klotho regulation [20]. Reduced phosphate excretion in CKD leads to phosphate retention, which can subsequently stimulate FGF23 secretion from osteocytes and osteoblasts in bone [21]. Elevated FGF23 can increase the urinary excretion of phosphate, limiting further hyperphosphatemia; however, sustained elevation of FGF23 can lead to the reduced synthesis of active vitamin D (calcitriol), which is the main stimulant for increased PTH secretion [22]. A side effect of PTH is the mobilization of calcium from bone, exacerbating mineral metabolism disturbances and promoting vascular calcification [23]. Our results confirmed higher blood levels of phosphate (in line with other original studies [24,25] and the conclusions of several reviews [26,27,28]), lower levels of vitamin D (in line with previous studies [29,30,31]), higher levels of PTH (in line with previous studies [32,33,34]), and non-significantly higher levels of calcium (in line with previous studies [35,36,37]) in the CKD group compared with the CTL and SH groups. In addition, we observed higher serum levels of FGF23 in the CKD group compared with the CTL and SH groups, which is consistent with previous studies [38,39,40,41].

Klotho deficiency is another common symptom in CKD [42]. Klotho binds to the FGF receptor, enhancing the receptor’s affinity and specificity for FGF23. This Klotho–receptor complex is essential for effective FGF23 signal transduction in canonical target tissues such as the kidney. Klotho could also help in preventing calcium loss in the urine and maintaining normal serum calcium levels [43]. Moreover, Klotho can influence PTH secretion both directly and indirectly, further affecting mineral metabolism [44]. Reduced Klotho levels have been reported continuously in CKD [42,45,46], in line with our results.

In CKD patients, elevated FGF23 is associated with a higher prevalence and incidence of AF, with some of these effects resulting from left ventricular hypertrophy, atrial enlargement, and heart failure events [4], as confirmed in our study (higher heart weight and heart-to-body weight ratio in the CKD group compared to the CTL and SH groups). Experimental models of CKD have shown that increased circulating FGF23 binds to cardiac FGFR4, activating pathways such as AKT that promote AF and electrical conduction abnormalities [5]. Klotho deficiency exacerbates this process by disrupting the regulation of FGF23 signaling, aggravating disturbances in mineral metabolism and cardiac remodeling and consequently increasing the risk of AF in CKD [5].

Furthermore, our results showed lower and higher heart levels of Bcl2 and BAX in the CKD group compared with the CTL and SH groups, respectively. Hypertrophy and increased apoptosis, along with observed AF, may indicate that hypertrophic cells typically experience metabolic and contractile dysfunction, contributing minimally to effective contraction. Apoptosis, however, selectively removes living and functioning cardiomyocytes [47]. However, these results are more correlational than causative. Increased heart apoptosis in response to CKD has been documented in previous studies [48,49].

Exercise improves kidney and vascular function, which can in turn improve phosphate handling, leading to decreased levels of FGF23. In addition, exercise can reduce systemic inflammation and increase phosphate clearance [15]. Exercise also improves calcium regulation; all of these effects could help to reduce FGF23 levels in CKD [15]. Our results showed decreased FGF23, which was corelated with decreased phosphate and a non-significant reduction in blood calcium levels. Previous studies have reported similar results [50]. However, another study reported that lying exercise did not affect FGF23 levels. Based on previous studies [51,52], exercise should stress bone and regulate calcium turnover to affect FGF23. This may explain why this study did not observe a significant effect on FGF23. A study by Falahi et al. [53] did not observe a reduced phosphate load after exercise in CKD, which could have been due to the different exercise protocol compared with ours.

Both types of exercise also decreased the PTH level, which could reflect improved calcium regulation due to exercise [54]. Ramezanipoor et al. [55] and Ebrahim et al. [56] reported similar results, as did other studies [57,58,59]. We also observed higher levels of vitamin D in the blood, associated with reduced PTH.

In addition, by improving tubule function [60,61], exercise could increase the production and secretion of Klotho. However, we did not measure tube function directly. Previous studies have also reported increased plasma levels of Klotho after exercise [62,63].

Additionally, exercise decreased heart BAX levels and increased Bcl2 levels. Previous studies showed that exercise activates the PI3K/Akt pathway, which phosphorylates and inactivates pro-apoptotic Bad (allowing Bcl2/Bcl-xL to suppress BAX translocation to mitochondria) while upregulating Bcl2 transcription via Nrf2 or reduced oxidative stress [64,65]. It also lowers CKD-induced inflammation (e.g., TNF-α, IL-6), suppressing extrinsic apoptosis signals like Fas/caspase-8 that amplify mitochondrial BAX. It should be noted that we did not evaluate the levels of the molecules involved in mediating exercise’s effects on apoptosis, instead focusing only the final molecules (BAX and Bcl2). Previous studies have confirmed that exercise improves the cardiac apoptosis balance in CKD models [66]. Previous studies have also shown that exercise improves apoptosis [67,68].

Finally, our functional results showed reduced AF susceptibility (i.e., shorter pacing-induced AF duration) and heart function in the exercise groups, consistent with other studies [69,70]. Mechanistically speaking, with the reduced accumulation of phosphate and calcium, the accumulation of phosphocalcium could be decreased, leading to reduced AF susceptibility, shorter induced AF episodes, and improved heart function. Additionally, Klotho may reduce TGFβ1 and WNT signaling, further improving AF and heart function [71]. However, our study was correlational, and we also failed to measure TGFβ1 and WNT.

## 5. Limitations

Mechanistic studies investigating BAX/Bcl-2 signaling pathways and vitamin D regulatory mechanisms were not possible in the current research due to financial constraints. Additionally, there was a lack of renal histological analysis. While the 5/6 nephrectomy rat model effectively recapitulates CKD-induced FGF23/Klotho dysregulation, mineral disorder, and AF susceptibility observed in patients, its acute induction in otherwise healthy young rats diverges from typical human CKD trajectories (gradual progression over years with comorbidities). This could also limit the tolerability of HIIT in human patients with CKD. The small sample size may also limit the generalizability of these results. Gender could also affect the results, as sex hormones play key roles in these processes. Therefore, we recommend future mechanistic studies with a larger sample size including both genders and a pathological analysis of renal tissue.

## 6. Conclusions

Both HIIT and aerobic training were associated with attenuated AF susceptibility and improved atrial electrophysiological properties, elevated serum FGF23, reduced Klotho, increased BAX, and a prolonged P-wave duration. HIIT was superior to continuous aerobic training in reducing heart weight, BAX, and the P-wave duration, while the effects on other biomarkers were comparable.

## Figures and Tables

**Table 1:** Aerobic training program.

	Week 1	Week 2	Week 3	Week 4	Week 5	Week 6	Week 7	Week 8
Velocity (%Vmax)	45	45	45	45	45	50	50	55
Slope (º)	0	0	0	1	1	1	1	1
Duration (min)	16	20	25	27	30	30	35	35
Distance covered in each session	240	300	375	430	480	480	600	600
Distance covered in one week	720	900	1125	1300	1440	1440	1800	1800

**Table 2:** High-intensity interval training program.

	Week 1	Week 2	Week 3	Week 4	Week 5	Week 6	Week 7	Week 8
High-intensity interval velocity (%Vmax)	80	80	85	85	90	90	95	100
High-intensity interval duration (min)	1	1	1	1	1	1	1	1
Low-intensity interval velocity (%Vmax)	40	45	45	50	50	55	55	55
Low-intensity interval duration (min)	1.5	1.5	1.5	1.5	1.5	1.5	1.5	1.5
Total duration of one session (min)	30	30	30	30	30	30	30	30
Distance covered in each session (m)	240	300	360	420	480	480	540	540
Distance covered in one week (m)	720	900	1080	1260	1440	1440	1620	1620

## Data Availability

The original contributions presented in this study are included in the article/Supplementary Materials. Further inquiries can be directed to the corresponding author.

## Supplementary Materials

The following supporting information can be downloaded at https://www.mdpi.com/article/10.3390/biom16040513/s1. Supplementary Table S1: Vmax of two training groups across various weeks; Supplementary Table S2: The results of Shapiro-Wilk and Leven tests confirmed normality and homogeneity of variance for all the variables.
