# Supplementary material for: High-Intensity Interval and Aerobic Training Alleviate Cardiac Pathology, Apoptosis, and Atrial Fibrillation in Rats with Chronic Kidney Disease: The Roles of FGF23 and Klotho
Source: Biomolecules. 2026 Mar 30;16(4):513. doi: 10.3390/biom16040513 (PMC13113790; doi:10.3390/biom16040513)
Supplement: Supplementary file 1 [file biomolecules-16-00513-s001.zip › biomolecules-4197306-supplementary.pdf]

Supplementary Materials

Supplementary Table S1: Vmax of two training groups across various weeks.

CKD+HIIT

	Week1	Week2	Week3	Week4	Week4	Week6	Week7	Week8
Vmax	28	32	34	36	36	38	38	40

CKD+MICT

	Week1	Week2	Week3	Week4	Week4	Week6	Week7	Week8
Vmax	30	32	32	34	34	36	36	38

Supplementary Table S2: The results of Shapiro-Wilk and Leven tests confirmed normality and homogeneity of variance for all the variables.

Variable	Shapiro-Wilk p	Levene's p	η^2 Effect Size
Body weight	0.214	0.772	0.05
Heart weight	0.187	0.321	0.79
Heart/Body ratio	0.156	0.367	0.79
FGF23	0.089	0.482	0.98
Klotho	0.123	0.241	0.79
Vitamin D	0.067	0.619	0.88
Phosphate	0.098	0.162	0.62
Calcium	0.234	0.851	0.53
PTH	0.145	0.419	0.76
BAX	0.112	0.468	0.82
Bcl2	0.178	0.282	0.79
BAX/Bcl2	0.076	0.559	0.86
P-wave duration	0.134	0.338	0.79
